# Selection and validation of endogenous reference genes using a high throughput approach

**DOI:** 10.1186/1471-2164-5-55

**Published:** 2004-08-13

**Authors:** Ping Jin, Yingdong Zhao, Yvonne Ngalame, Monica C Panelli, Dirk Nagorsen, Vladia Monsurró, Kina Smith, Nan Hu, Hua Su, Phil R Taylor, Francesco M Marincola, Ena Wang

**Affiliations:** 1 Immunogenetics Section, Department of Transfusion Medicine, Clinical Center, NIH Bethesda, MD 20892, USA; 2 Biometric Research Branch, Division of Cancer Treatment and Diagnosis, Cancer Prevention Studies Branch (CPSB), National Cancer Institute, National Institutes of Health, Bethesda, MD 20892, USA

## Abstract

**Background:**

Endogenous reference genes are commonly used to normalize expression levels of other genes with the assumption that the expression of the former is constant in different tissues and in different physiopathological conditions. Whether this assumption is correct it is, however, still matter of debate. In this study, we searched for stably expressed genes in 384 cDNA array hybridization experiments encompassing different tissues and cell lines.

**Results:**

Several genes were identified whose expression was highly stable across all samples studied. The usefulness of 8 genes among them was tested by normalizing the relative gene expression against test genes whose expression pattern was known. The range of accuracy of individual endogenous reference genes was wide whereas consistent information could be obtained when information pooled from different endogenous reference genes was used.

**Conclusions:**

This study suggests that even when the most stably expressed genes in array experiments are used as endogenous reference, significant variation in test gene expression estimates may occur and the best normalization is achieved when data from several endogenous reference genes are pooled together to minimize minimal but significant variation among samples. We are presently optimizing strategies for the preparation of endogenous reference gene mixtures that could yield information comparable to that of data pooled from individual endogenous reference gene normalizations.

## Background

Endogenous reference alse referred to as house keeping genes defines in biology the theoretical assumption that certain genes are ubiquitously expressed in nucleated cells possibly because their stable expression is essential for cell survival and welfare in all physio-pathological circumstances. In practical terms, endogenous reference genes provide a useful constant reference to normalize the expression of test genes in different tissues and in different conditions. This is obviously important when estimates of gene expression are provided in relative terms rather than absolute units of measurement. Thus, endogenous reference genes are used as common denominator in biological fractions where the expression of a test gene is described as the relative ratio over an arbitrarily selected internal control presumed to be stably expressed in all circumstances relevant to the experiment [1-3]. Most frequently, glyceraldehydes-3-phosphate dehydrogenase (GAPDH) [4,5], albumin (for hepatocytes) [6], β-, γ-actins [7,8], cyclophilin [9,10], α-, β-tubulins [7,11], hypoxantine phosphoribosyltransferase (HRPT) [12,13], L32 [14,15] and 18S, 28S ribosomal RNA (rRNA) [16-18] have been used as endogenous reference genes. Depending upon the experimental design, endogenous reference genes have been used individually or in combination for Northern blot analysis, reverse transcription polymerase chain reaction (RT-PCR) and quantitative real-time PCR (qRT-PCR) analysis [19,20]. With the development cDNA microarray technology endogenous reference genes have been used for array data normalization. However, accumulation of extensive data bases suggests that the expression of frequently used endogenous reference genes can vary substantially according to materials and conditions studied [1,2,6,14,17,18,20-27].

Powerful insights in patterns of gene expression could be attained recently through cDNA or oligonucleotide-based global transcript analysis tools that apply a constant reference system to determine ratios of gene expression across large data sets [28,29]. The constant reference is provided for each gene in question by consistently co-hybridizing individual test samples with a differentially labeled reference sample maintained identical throughout all the hybridization experiments. Gene expression data are then expressed as the ratio of expression between test and reference samples for each gene. By keeping the reference sample identical the resulting ratio represents a precise estimate of the relative expression of each gene across the various conditions tested bypassing the need to normalize with endogenous reference genes. This holds true if the hybridization kinetics between test and reference sample are accurately reproducible. We will refer to this concept as "reference concordance" and in the results we will discuss how reference concordance was used to validate the reproducibility of cDNA array data from which putative candidate endogenous reference genes were identified.

In the present study, we tested a set of 419 consecutive experiments performed on a 17,000 gene cDNA array platform to which RNA from neoplastic or normal tissues were consistently co-hybridized with a differentially-labeled reference RNA derived from peripheral blood mononuclear cells (PBMC) pooled from six normal donors. The following steps were pursued: 1) Reproducibility assessment of the data set through determination of reference concordance. This was achieved by repeating 14 reference experiments using the melanoma cell line A375 as test sample (Cy5) co-hybridized with pooled PBMC as reference (Cy3). To test for inter-array and printing variation, slide number one and every other 25 slides in sequential order of printing (100 slides per printing set) were used for the repetitive A375 / pooled PBMC hybridizations. In addition, to assess labeling bias, reciprocal labeling was alternatively applied as previously described [30]. In this fashion a pool of genes expressed with high level of reference concordance was selected. 2) Identification of putative endogenous reference genes was performed on 384 array experiments of unequivocal quality by selecting genes that had demonstrated high reference concordance (>90% of the genes in the arrays) and ranking them from the lowest to the highest variance of _Log2 _test / reference ratios across all array experiments. 3) Validation of the candidate endogenous reference genes as predictor of relative gene expression in large data series. For this purpose, we tested the relative estimates of expression of the melanocytic lineage-restricted melanoma differentiation antigen gp100/Pmel17 (gp100) [31] in melanocytic and non-melanocytic tissues. Estimates of expression of gp100 were compared after normalization with different endogenous reference genes. For this analysis, we used a new tool that spots cDNA libraries from different tissues on an array platform to allow high-throughput evaluation of individual gene expression across broad tissue collections. We termed this tool: "transcriptome array". 4) Validation of endogenous reference gene-based normalization of gene expression by qRT-PCR and protein expression.

## Results

### Data processing

The GenePix Pro 4.0 software was used for array image analysis and calibration. All statistical analyses were performed with the SPLUS package . Thirteen arrays with missing data in more than 30% of the spots and 8 arrays with irregular distribution according to M-A plots (M and A representing respectively log-ratios and average intensities) [32] were excluded. Spots in which > 50% of the pixels reached saturation in either channel, flagged spots and spots with intensity ≤ 200 in one channel and ≤ 500 in the other channel were filtered out. If the intensity in one channel was < 200 but that of the other channel was > 500, we arbitrarily assigned an intensity of 200 to the channel with the lowest intensity. The _log2_Cy5/Cy3 ratios were normalized by approximating median values to zero. Spots with ratios > 100 or < 1/100 were truncated at 100 and 1/100 respectively. Data normalization was done by median centering _Log2_ratio of all genes for each array. For data normalization we used certain arbitrary cutoff criteria to remove spots with weak signal. Weak signal approximating background fluorescence is not reliable and the corresponding log ratios are often distorted resulting in disproportionately high or low values that would bias the statistical results. Because the interpretation of spots with low signal is difficult to make we have adopted the policy of excluding them to focus the analysis on genes whose expression pattern is more reliably tracked by the array tool. In regard to spots with a ratio of >100 or <-1/100, most of them are due to extremely weak signal in one channel which generates a disproportionately extreme value. Although there is no published "gold standard" for the selection of such cutoffs, in practice the parameters that we used for this study are most commonly accepted by investigators and reasonable because allow retention of most of the data in the array excluding only the most extreme and least reliable information.

### Validation of the data set through analysis of "reference concordance"

To select from the data base the most reliable genes, we first assessed reproducibility by testing the level of concordance in 14 reference experiments. Reference concordance relies on the expectation that results obtained by repeatedly hybridizing the same test and reference material should perfectly collimate and the degree of deviation from such prediction estimates experimental variance. Concordance can be easily measured by periodically re-hybridizing the same test sample with the constant reference sample. We analyzed a matrix of 7 forward and 7 reciprocally-labeled replicate array experiments hybridized periodically every other 25 cDNA array slides. Reciprocal labeling was applied to measure labeling bias [30]. Data generated by reciprocal experiments were mathematically reversed into the same labeling direction for data analysis. Genes that were discordant due to labeling bias were identified by student's *t *test as those with ratios significantly different between the 7 forward and 7 reciprocal experiments after reversal of the reciprocal values (P < 0.05) and the median ratio difference between the 7 forward and 7 reciprocal experiments was larger than 1.5 fold. The genes whose variances of the log ratio across all experiments were among the top 1 percentile of variance of all genes were identified as discordant due to hybridization bias. In addition, genes with more than 50% missing values were excluded. Overall 1,343 out of 16,738 were judged potentially discordant and were excluded from further analysis. The remaining 15,395 concordant genes were used for subsequent analyses.

### Endogenous reference gene identification

We studied 384 of 419 consecutive array experiments remaining after the exclusion of the 14 reference concordance experiments and 21 experiments judged of poor quality due to missing data or irregular distribution by M-A plot analysis. Selection of candidate endogenous reference genes followed these steps.

First, the seven experiments used for the analysis of reference concordance in which labeling was done identically to the rest of the experimental samples (test labeled with Cy5 and reference with Cy3) were used to calculate experimental variance (Table 1). Median of variances across the seven replicate arrays was calculated for all genes with average intensity in both channels > 2,000. This parameter provides an estimate of the variance due to experimental variation (background variance) since theoretically no differences in gene expression should be noted by using the same material. Based on the assessment of the seven repeat experiments, we defined differential expression as > 2 standard deviations (SD) from the mean of the 384 arrays which is equivalent to 1.46-fold change (± 0.549 in _log2 _ratio). Consequently, genes with fold changes < 1.46 from zero across all 384 arrays were categorized as candidate endogenous reference genes and were ranked according to ascending values of SD of _Log _ratio (Table 1).

**Table 1:** 

				**384 experiments**		**7 replicates**	
				
**Gene Name**	**Image ID**	**Unique ID**	**Mean Intensity**	**Mean of logRatio**	**SD of logRatio**	**Mean of logRatio**	**SD of logRatio**
cDNA FLJ40458	1571492	Hs.181346	2835	0.19	0.27	0.39	0.15
Unknown	301067		2571	0.1	0.27	0	0.21
PTH	322051	Hs.37045	3380	0	0.28	-0.02	0.27
KIAA1935	39938	Hs.300776	2359	0.16	0.28	0.01	0.19
ESTs	49313	Hs.395460	2079	0.15	0.28	0.08	0.03
cDNA FLJ30539 fis	49463	Hs.21489	2561	0.09	0.28	0.17	0.13
SDCCAG16	1576228	Hs.271926	2111	0.11	0.29	0.28	0.14
clone IMAGE:41799	278673	Hs.271721	3132	0.16	0.3	-0.04	0.1
PSIP2	289945	Hs.82110	2521	-0.07	0.3	-0.06	0.19
ETV2	1468722	Hs.194061	2646	0.09	0.31	0	0.28
ESTs	1571401	Hs.126999	2659	0.05	0.31	-0.04	0.08
NEDD8	277660	Hs.75512	3875	0.08	0.31	0.1	0.2
HIP2	486259	Hs.155485	2511	0.11	0.31	0.12	0.25
HIRIP5	745314	Hs.430439	3407	-0.08	0.31	0.07	0.31
ACTR8	156363	Hs.124219	2731	0.08	0.32	0.14	0.16
GPLD1	293696	Hs.272529	3110	-0.03	0.32	-0.05	0.28
KIAA0769	299128	Hs.19056	2148	0.14	0.32	0.05	0.2
CSNK1G2	346031	Hs.181390	2320	-0.03	0.32	-0.24	0.28
RAB31	784150	Hs.223025	3479	-0.06	0.32	-0.36	0.36
C14orf117	796100	Hs.103189	2008	0.12	0.32	-0.01	0.16
ATP6IP2	825077	Hs.183434	2159	0.02	0.32	0.28	0.32
SNRPD3	897099	Hs.1575	3031	-0.04	0.32	-0.48	0.25
Unknown	1292073		2745	-0.13	0.33	0.05	0.28
ESTs	22137	Hs.187406	3133	0.07	0.33	-0.01	0.21
ESTs	32782	Hs.443140	2464	-0.02	0.33	0.09	0.26
L3MBTL	43090	Hs.300863	2581	-0.08	0.33	-0.41	0.31
GNRH1	487071	Hs.82963	3294	0.05	0.33	0.23	0.23
clone 24629 mRNA	746258	Hs.142570	2645	0.05	0.33	-0.21	0.14
FLJ12998	826367	Hs.343627	2267	0.14	0.33	0.03	0.3
cDNA: FLJ23477 fis	1492329	Hs.145362	2142	0.09	0.34	0.35	0.34
HOXC4	1756945	Hs.50895	2581	0.03	0.34	-0.02	0.22
WSX1	1855887	Hs.132781	3259	-0.01	0.34	0.12	0.3
HIST1H1A	1872543	Hs.150206	2523	0.07	0.34	-0.17	0.14
TFAP2B	363144	Hs.33102	2510	0.05	0.34	0.67	0.35
TRIM31	509760	Hs.91096	2753	0.06	0.34	-0.08	0.2
FLJ00166	713191	Hs.43213	3134	0.08	0.34	0	0.22
CDK5R1	757873	Hs.93597	2965	-0.12	0.34	-0.22	0.15
PPP3CA	796730	Hs.272458	2605	-0.07	0.34	-0.36	0.16
PMS2L4	161373	Hs.278468	2534	-0.14	0.35	-0.21	0.22
DLGAP1	1758491	Hs.75814	2143	0.02	0.35	-0.24	0.2
ESTs	177884	Hs.14613	2054	0.01	0.35	-0.22	0.25
NRL	2364249	Hs.89606	2300	-0.04	0.35	-0.09	0.14
RAB36	281489	Hs.38772	3143	-0.08	0.35	0	0.12
RPC8	323603	Hs.353192	2367	-0.06	0.35	-0.33	0.24
ZNF177	33294	Hs.172979	2988	0.01	0.35	-0.21	0.25
LY6G5C	448136	Hs.246845	3591	-0.02	0.35	-0.44	0.28
RAD17	586844	Hs.16184	3797	-0.01	0.35	0.12	0.15
LOC253039	1033388	Hs.41181	3612	-0.02	0.36	0.31	0.08
IMPDH2	1582050	Hs.75432	2709	-0.01	0.36	-0.08	0.18
PRMT3	2074202	Hs.152337	2042	0.07	0.36	-0.06	0.21
FZD6	214916	Hs.114218	3884	0.03	0.36	0.02	0.26
AP3B2	47510	Hs.21022	2350	0.03	0.36	-0.23	0.31
NF2	769716	Hs.902	2887	-0.08	0.36	-0.11	0.31
SCP2	855395	Hs.75760	3218	-0.12	0.36	-0.1	0.19
COL8A1	1472775	Hs.114599	4178	-0.06	0.37	0.05	0.18
AKAP4	1643144	Hs.97633	2557	-0.03	0.37	-0.07	0.15
Unknown	1671546		3386	0.03	0.37	1	0.42
TCF8	178463	Hs.232068	2726	-0.04	0.37	-0.01	0.06
TNP2	1839038	Hs.2748	2737	0.08	0.37	0.11	0.24
PIWIL1	2329739	Hs.194712	2573	-0.08	0.37	-0.14	0.22
DBCCR1	47037	Hs.6090	2481	-0.01	0.37	-0.18	0.24
SLC21A3	289706	Hs.46440	2708	0	0.38	-0.11	0.27
ESTs	35105	Hs.403854	3126	0.03	0.38	0.23	0.18
cDNA FLJ34400 fis	511835	Hs.380035	3058	0	0.38	0.02	0.37
CNTN1	51640	Hs.143434	2479	-0.05	0.38	-0.11	0.3
TFCP2	843067	Hs.154970	3728	-0.06	0.38	0.16	0.34
C17orf35	510032	Hs.15196	2778	-0.02	0.39	0.09	0.28
AFAP	488062	Hs.80306	3142	0	0.42	0.12	0.15
MTP	731054	Hs.195799	2764	-0.01	0.42	-0.09	0.4

Among these genes, we further selected candidate endogenous reference genes according to the following criteria: in at least 95% of the 384 arrays the _Log2 _ratio had to be within 2 SD from zero and the average intensities of both channels across all samples need to be higher than 2,000. The intensity parameter was added to ensure that the selected endogenous reference genes were expressed at a relatively high level in most tissues to mitigate excessive fluctuations in _Log2 _ratios occurring when a low value is applied as denominator. This is important when a reference gene is applied as a denominator in the equation used to normalize the ratio of other tests genes; in such cases robust gene expression decreases the range of ratios resulting from the analysis decreasing, therefore, the experimental variance. Sixty-nine genes fit these criteria. The range of mean _Log2 _ratio was from 0 to 0.15 and the SD of mean _Log2 _ratio was from 0.27 to 0.42. Analysis of 7 replicate array based on the same group of candidate endogenous reference genes demonstrated a similar distribution of mean _Log2 _ratio and SD of mean _Log2 _ratios (with exception of TFAP2B) suggesting that this variance could be attributed to predominantly experimental noise. Among these genes we further selected for validation 11 that had high mean fluorescence intensity (underlined in Table 1). However, probe preparation and other technical considerations limited to analysis to only 8 of these genes which included: NEED8, HIRIP5, GPLD1, RAB31, SNRPD3, FZD6, COL8A1 and AFAP.

"Leave-one-out cross-validations" were used to estimate the error [33,34]. In each validation experiment one array was left out. The remaining 383 arrays were used to identify endogenous reference genes using the same parameters used for the original analysis (test/reference ratios < 2 SD in at least 95% of the experiments and average intensity > 2,000 in both channels). The median _Log2 _ratio of the endogenous reference gene in the left out array was then compared with the "real" normalization factor consisting of the median _Log2 _ratio for all genes in that array supposed to approximate a balanced expression of test and reference genes for that array. This procedure was repeated for each array 384 times. Only in 3 arrays out of 384 (2.3%) were found errors in endogenous reference gene selection (errors were considered median _Log2 _ratio of endogenous reference genes in the left out array > 2 standard deviations apart from the "real" normalization factor in the same array (which is zero for normalized arrays).

The expression of each candidate endogenous reference gene identified in this study was then compared with available information about the expression of the same genes in 12 normal human tissues reported by the Affymetrix HG-U95A-E probe sets . We found a good correlation between the expression patterns observed by us and that reported by the Affymetrix GeenChip array (data not shown). In addition, a cluster of endogenous reference genes suggested by Applied Biosystems for standardization during qRT-PCR was analyzed by evaluating the SD of their Log2 ratios across the 384 arrays. As data indicated in Table 2, although some genes (bold) demonstrated mean SD _Log2 _ratios comparable to that of the endogenous reference genes identified in this study, the corresponding percentage of the arrays in which _Log2 _ratios were within 2 SD from zero ranged between 0.68–0.85 which is significantly lower than the frequency in which the same parameter fell for the 69 candidate house keeping genes identified here (>95%). In addition, we looked at the variation in our arrays of genes classically used as endogenous reference genes including: ribosomal protein L32, HRPT, β-actin and tubulin-α3. In all cases the mean SD of _Log2_Ratio was relatively larger than for the endogenous reference genes identified in Table 1 with the following respective values: 0.88 for Ribosomal protein L32, 1.13 for HRPT, 1.32 for β-actin and 1.42 for laminin-α3. Interestingly, however, the mean intensities were relatively higher for all of these genes compared with the ones identified in this study (7,369; 12,778, 43,794 and 42,241 for the four genes respectively) suggesting that these genes are expressed probably at higher concentrations in tissue and, therefore, although relatively variable in expression in different tissues, they have been a useful marker of RNA abundance when only rough estimates are required like, for instance, for Northern Blotting.

**Table 2:** 

**Gene Name**	**Image ID**	**Description**	**Mean Int**	**Mean logR**	**SD logR**	**% of arrays**
NKTR	712460	NK-tumor recognition protein=cy	7319.55	0.62	0.79	0.52
NKTR	712460	NK-tumor recognition protein=cy	3048.28	**0.19**	**0.43**	**0.84**
NKTR	712460	NK-tumor recognition protein=cy	8637.15	0.99	0.99	0.53
NKTR	2064497	natural killer-tumor recognitio	959.97	0.72	0.85	0.7
PPIC	882459	peptidylprolyl isomerase C (cyc	5034.82	2.11	1.45	0.44
PPIB	756600	peptidylprolyl isomerase B (cyc	6606.09	1.08	1.04	0.39
PPIL2	450661	peptidylprolyl isomerase (cyclo	3035.17	**0.16**	**0.4**	**0.85**
PPIL2	2017652	peptidylprolyl isomerase (cyclo	850.93	**0.23**	**0.48**	**0.76**
PPIG	809621	peptidyl-prolyl isomerase G (cy	3991.93	0.28	0.53	0.67
PPID	884500	peptidylprolyl isomerase D (cyc	5805.51	0.33	0.57	0.66
PPID	71154	peptidylprolyl isomerase D (cyc	5652.12	0.27	0.52	0.63
PPIH	767277	peptidyl prolyl isomerase H (cy	3277.26	0.28	0.53	0.73
PPIF	758343	peptidylprolyl isomerase F (cyc	15900.53	3.18	1.78	0.13
CYP2J2	454580	cytochrome P450, family 2, subf	2302.76	0.71	0.84	0.65
PPIA	2580290	peptidylprolyl isomerase A (cyc	25478.36	1.32	1.15	0.36
PPIA	1570861	peptidylprolyl isomerase A (cyc	5028.34	0.76	0.87	0.46
GAPD	50117	glyceraldehyde-3-phosphate dehy	38011.52	1.66	1.29	0.4
GAPD	530934	glyceraldehyde-3-phosphate dehy	10526.82	1.14	1.07	0.37
GAPD	530868	glyceraldehyde-3-phosphate dehy	5052.59	0.81	0.9	0.25
GAPD	755641	glyceraldehyde-3-phosphate dehy	1693.23	**0.17**	**0.42**	**0.84**
PGK1	949939	Phosphoglycerate kinase 1	4452.92	**0.16**	0.4	0.85
B2M	878798	beta-2-microglobulin	34211.05	2.08	1.44	0.43
AMBP	2063390	alpha-1-microglobulin/bikunin p	1813.96	**0.15**	0.39	0.86
GUSB	2273001	glucuronidase, beta	6205.14	0.81	0.9	0.41
GUSB	276449	glucuronidase, beta	2241.73	**0.22**	**0.47**	**0.68**
HPRT1	280507	hypoxanthine phosphoribosyltran	12778.31	1.29	1.14	0.35
TBP	280735	TFIID=TATA box binding protein	4065.4	**0.24**	0.49	0.73
TFR2	461750	transferrin receptor 2	2700.22	**0.19**	**0.44**	**0.84**
TFR2	2408681	transferrin receptor 2	2958.88	**0.21**	**0.46**	**0.82**

### Test gene expression estimates according to house-keeping gene selection: the transcriptome array

To validate the usefulness of candidate endogenous reference genes in large sample populations we developed a new tool that we are planning to use in the future for validation of gene expression across extensive data sets. This tool displays cDNA libraries originated from different tissues or cell lines individually spotted on a solid surface. The principle of this technology is similar to RNA dot blot which uses RNA isolated from samples and transferred to membrane making, therefore, the transcriptome array a high-density dot blot. The labeled DNA probe of interest is hybridized to the immobilized complementary strain of mRNA. A reference gene hybridization will carried out simultaneously to estimate the relative expression of the gene of interest compared with the reference gene. The new tool we describe here, termed transcriptome array, utilizes cDNA generated from source mRNA for target immobilization to improve the stability of the immobilized targets and differentially fluorescence-labeled test and reference probes (RNA or double strained DNA) then can co-hybridize on to the same spots. Using a validated RNA amplification technology [30], large quantity of pure amplified RNA with proportional representation of source mRNA species could be generated from which cDNA could be obtained through a reverse transcription reaction. Because of the minimum amount of cDNA used for fabricating each transcriptome array (<5 nano gram cDNA/spot) and the size of spots (100 um), the expression of a large number of genes can be theoretically analyzed on thousands of different samples simultaneously. Since the amount of cDNA spotted may vary according to the quality of the starting material and the efficiency of RNA preparation for each sample, absolute estimation of fluorescence from the hybridized probe is not informative of the expression of the given gene in each sample. Therefore, a reference system is necessary so that comparative expression of the test gene can be presented proportional to that of a consistently expressed gene across all samples. Therefore, interpretation of data derived from the transcriptome array relies on endogenous reference gene normalization.

To test the usefulness of various endogenous reference gene we resorted to the well characterized expression of the melanocytic lineage-specific gene gp100/Pmel17 [31] that is expressed exclusively though heterogeneously in cells of melanocytic lineage [35,36]. The assumption of this experiment was that in spite of its heterogeneity of expression in samples of melanocytic origin, overall the expression of gp100 should be higher in meloanocytic compared with non melanocytic samples with a high degree of significance. Rho-C has been associated with the metastatization process in melanoma but its specificity for melanocytic lineage is unknown [37]. The differentiation control element DICE is found in the 3'-UTR of numerous eukaryotic mRNAs and there is no solid association between its pattern of expression and specific physio-pathological or developmental conditions [38]. Therefore, we compared the expression profile of these three genes in 829 cDNA libraries that included 106 melanoma cell lines, 127 melanoma metastases, 2 benign nevi (total of 235 melanocytic samples) and 593 miscellaneous samples containing a predominance of primary esophageal, renal and colon cancers paired with normal tissues from the same organ and a large number of circulating mononuclear cells. In addition, samples from most other normal and cancerous tissues were included although in smaller number (complete list of samples available upon request).

**Figure 1:**
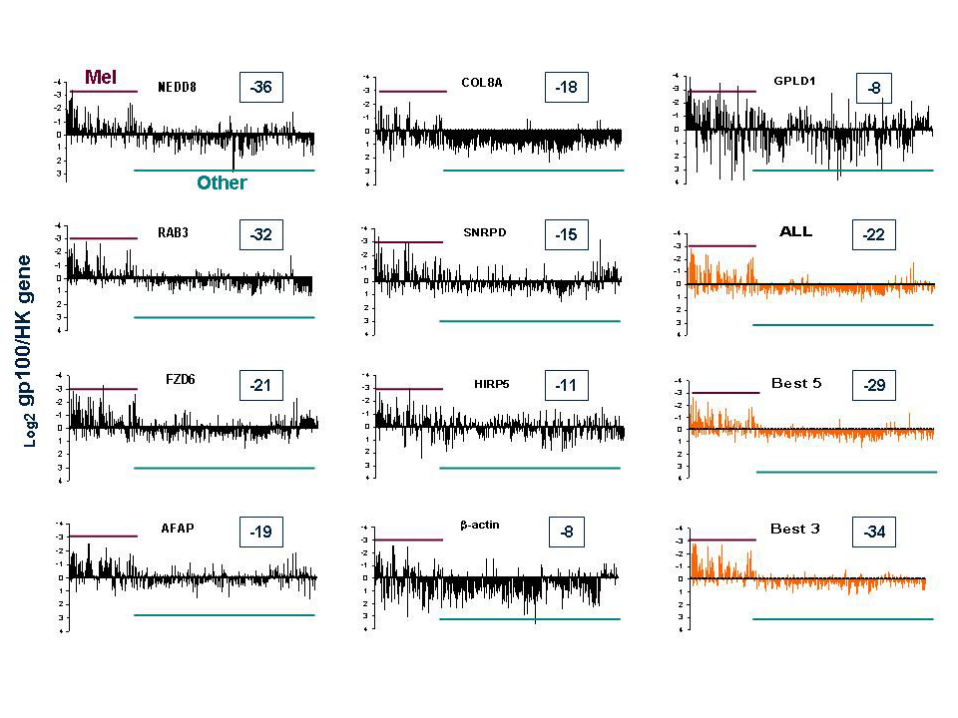
Comparison of gp100 expression normalized by different endogenous reference genes. Candidate endogenous reference genes were selected among those in Table 1. The gp100 probe (Cy3) was co-hybridized with one endogenous reference gene probe (Cy5) at the time to the transcriptome array. The transcriptome array included 235 cDNA libraries derived from samples of melanocytic lineage (maroon bar) and 594 cDNA libraries from samples of non melanocytic lineage (green bar). Melanocytic samples consisting in the overwhelming majority of melanoma metastases or melanoma cell lines while non-melanocytic libraries included a large collection of esophageal, kidney, colon and other cancers and several normal tissues or circulating mononuclear cells. The complete list of samples is available upon request. The expression of gp100 normalized with different endogenous reference genes was compared by unpaired two-tailed student *t *test and the endogenous reference genes were ranked according to the level of significance in their ability to discriminate between melanocytic and non-melanocytic lesions (data presented as the _Log10 _p_2_-value (shown in the boxes associated with individual graphs; for details see Figure 2). The distribution of the _Log2 _ratios for each individual cDNA library is shown for each gp100 / endogenous reference gene combination. In addition, results compiled using the average of the _Log2 _ratios for all the endogenous reference genes, the 5 and the 3 with the lowest p_2_-value are presented (orange bar graphs).

**Figure 2:**
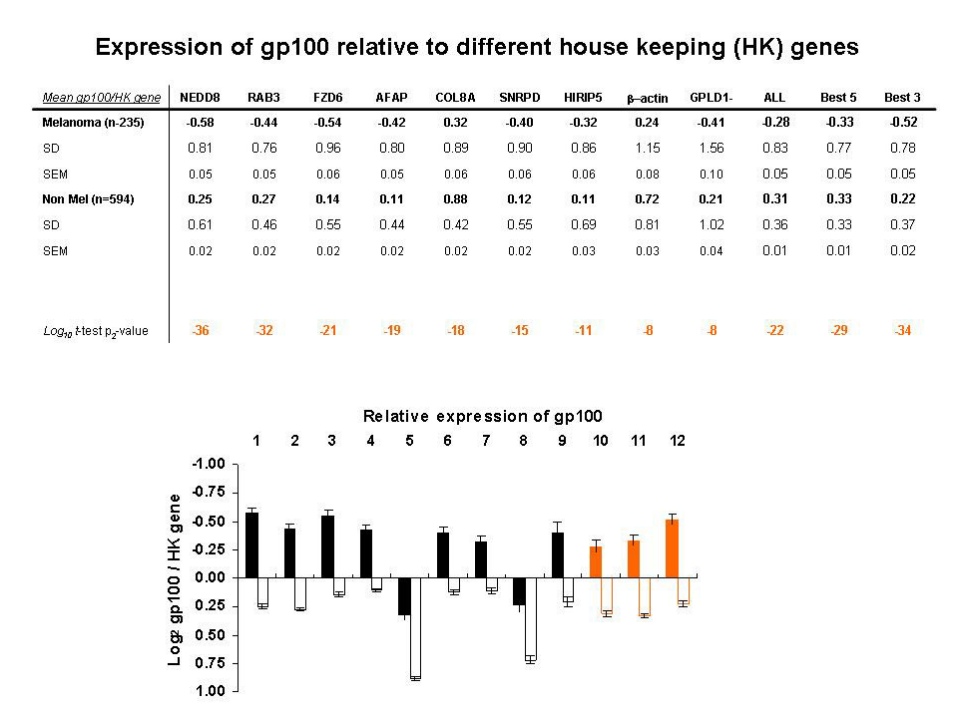
Expression of gp100 by melanocytic and non melanocytic lesions normalized with different endogenous reference genes. Average gp100 / endogenous reference gene _Log2 _ratios for melanocytic and non-melanocytic lesions are shown together with the standard deviation (SD) standard error from the mean (SEM) and the Log_10 _of the *t*-test p_2_-values when melanocytic and non-melanocytic lesions were compared. Also data derived from mathematically averaging the results obtained with all the endogenous reference genes, the ones yielding the best 5 individual p_2_-values and the best three are shown. The same data are presented visually in the bar graph below; in black are data derived with individual endogenous reference gene normalizations, in orange data derived by averaging results from different endogenous reference genes. Filled bares portrait data from melanocytic lesions, empty bars from non-melanocytic lesions.

As endogenous reference genes we chose β-Actin and new candidate genes identified by this study (see previous section). The test genes (gp100, Rho-C and DICE) were separately hybridized to the transcriptome array. Each gene labeled with Cy3 was co-hybridized with individual endogenous reference genes labeled with Cy5. We then compared _Log2 _Cy5 / Cy3 ratios between melanocytic and non-melanocytic tissues. gp100 was, as expected, expressed more in melanocytic lesions with high degree of significance no matter what gp100 / endogenous reference gene combination was used. The range of significance, however, varied extensively depending upon the gp100 / endogenous reference gene combination. This difference was considered representative of the ability of different endogenous reference genes to normalize for tissue-specific gene expression patterns (Figure 1). In details, all endogenous reference genes could segregate melanocytic from non melanocytic lesions with a high degree of significance (Figure 2; unpaired two sample *t *test p_2_-values ranged from 6 × 10^-8 ^to 3 × 10^-36^). There was, however, a big range in the discriminatory capacity among endogenous reference genes with NEDD8, RAB3 and FZD6 providing the highest predictive value (*t *test p_2_-value = 3 × 10^-36^, 1 × 10^-32 ^and 1 × 10^-21 ^respectively) and β-actin being one of the least reliable (*t *test p_2_-value = 1 × 10^-8^).

The large variation in the results obtained using different endogenous reference genes to normalize gp100 expression could have been due to a higher stability of the expression of some genes across all samples or to a differential expression of the endogenous reference genes themselves in melanocytic lesion. In the latter case, the better results obtained could be coincidental and not useful in other experimental situations. We, therefore, tested whether pooling results obtained with all endogenous reference genes independently of each predictive value in this controlled experimental situation could yield results as informative about gp100 pattern of expression as those obtainable with individual endogenous reference genes, particularly those that provided the best prediction. The advantage of this strategy is that it does not depend on previous knowledge of gene expression patterns for the selection of individual endogenous reference genes not applicable in conditions, in which the suitability of a gene for a given experimental situation is, contrary to gp100, is unknown. Thus, we compared the predictive value of the average of gp100 / endogenous reference gene _Log2 _ratios obtained with all endogenous reference genes and those obtained using the genes that provided the best five or the best three results (Figure 2). The use of pooled information from various endogenous reference genes appeared to stabilize the non-melanocytic _Log2 _ratios gp100 / endogenous reference. In fact, the SD of the_Log2 _ratios gp100 / endogenous reference among non-melanocytic samples derived by pooling endogenous reference gene results were significantly lower than the SD obtainable with any individual endogenous reference gene (F-test). The basis for this test was that non-melanocytic lesions uniformly should not express gp100 and, therefore, the _Log2 _ratios for any gp100 / endogenous reference gene combination should be constant resulting in very low SD. SD for non-melanocytic lesions were much lower using pooled endogenous reference genes for the normalization (Figure 2) and this was true at extreme levels of significance (F-test value for SD when all endogenous reference genes were used for normalization compared with individual endogenous reference were 0, 1.5 × 10^-9^, 0, 5.6 × 10^-7^, 9.0 × 10^-5^, 0, 0, 0 and 0 for NEDD8, RAB3, FZD6, AFAP, COL8A, SNRPD, HIRIP5, β-actin and GPLD1 respectively). Similar results were obtained when results from the best 5 and best 3 endogenous reference genes were pooled together. In addition, even if pooling data together did not provide the highest level of discrimination compared to the best results obtained with selected individual endogenous reference genes, the capacity of pooled data sets to discriminate gp100 expression between melanocytic and non melanocytic samples was still very high (2 × 10^-22^; 4 × 10^-29 ^and 1 × 10^-34 ^respectively when all, the best 5 or the best 3 endogenous reference gene results were pooled together). Interestingly, different endogenous reference genes provided not only different levels of discrimination but also provided different estimates of gp100 expression in melanocytic lesions with gp100 / endogenous reference gene _Log2 _ratio above (NEED8, RAB3, FZD6, AFAP, SNRPD3, HIRIP5, GPLD1) or below (COL8A1, β-actin) 0 (Bottom graph, Figure 2). This, of course, has no impact on the ability to characterize patterns of expression of different genes in different tissues but rather suggests that the latter group of endogenous reference genes is expressed at higher copy number, therefore, biasing the data toward its own fluorescence channel. Simple adjustment in probe concentration could easily solve this problem.

**Figure 3:**
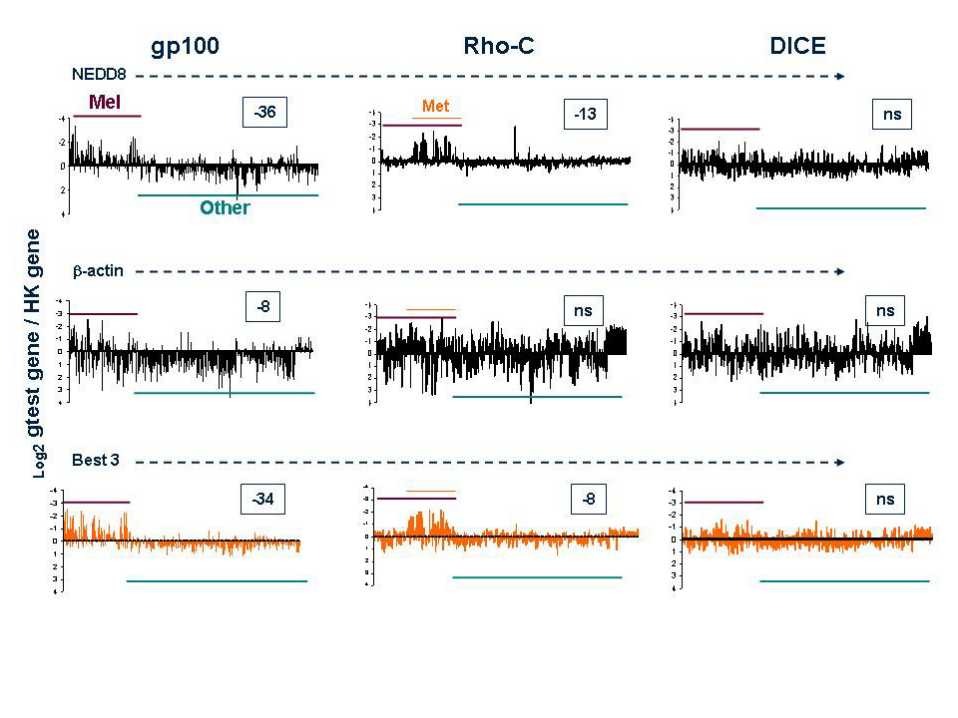
Relative expression of gp100, Rho-C and DICE in melanocytic and non-melanocytic samples. Test samples (Cy3) and endogenous reference genes (Cy5) were hybridized to the transcriptome array as described in Figure 1. Test gene / endogenous reference gene _Log2 _ratios are displayed as a bar graph for NEED8, β-actin and for the averages of test / endogenous reference gene_Log2 _ratios when the three endogenous reference genes with the best discriminating power in separating gp100 expression between melanocytic (maroon bar) and non-melanocytic (green bar) lesions (NEDD8, RAB3 and FZD6) were used. Samples derived from melanoma metastases are shown by the orange bar. In the boxes the _Log10 _p_2_-value of significance of differences between relevant samples (se text) are shown; ns = non significant).

We then analyzed the expression of Rho-C and DICE whose pattern of expression in the transcriptome array cannot be predicted based on available information save for the notion that of the expression of Rho-C is related to the metastatic process of melanoma [37]. The pattern of Rho-C was characterized with all the endogenous reference genes and representative information is presented in Figure 3. Most endogenous reference genes yielded a pattern suggestive of a preferential expression of Rho-C in melanoma metastases. In addition, averages of Rho-C / endogenous reference gene _Log2_ratios demonstrated clearly a specific expression of this gene in melanoma metastases. This pattern was not necessarily specific for melanoma as the other cancerous tissues spotted in the transcriptome array were obtained from primary lesions. Thus, it is possible that the preferential expression in melanoma metastases was due to the metastatic process rather than the melanocytic lineage. Interestingly, the melanoma cell line Mel-A375 did not constitutively express Rho-C as previously observed by Clark et al. [37]. In addition, none of the melanoma cell lines mostly derived from melanoma metastases demonstrated expression of Rho-C. This information suggests that Rho-C may be involved in the natural metastatic process *in vivo *but is not constitutively expressed *in vitro*. Finally, DICE did not demonstrate any specificity of expression and appeared variably expressed in all specimens independently of the endogenous reference gene used for normalization.

### Validation of data by protein analysis and quantitative real-time PCR (qRT-PCR)

**Figure 4:**
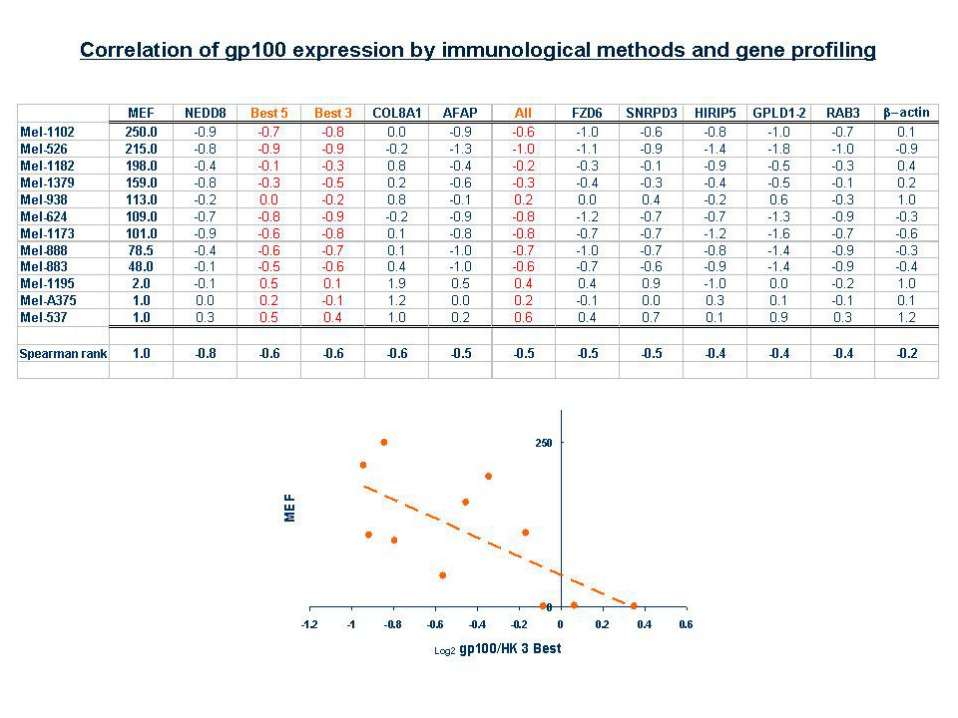
Correlation of gp100 expression estimated obtained by intracellular FACS analysis and by gene profiling with the transcriptome array. Cell lines with different level of gp100 expression by intra-cellular FACS analysis using the gp100-specific mAb HMB45 [35] were used for the analysis. The compiled information from the transcriptome array in which cDNAs from each cell line was spotted are shown. The Cy5/Cy3 (endogenous reference gene / gp100) _Log2 _ratios are shown for each endogenous reference gene evaluated. In addition, ratios derived by using the data from all the endogenous reference genes, the best 5 and best 3 according to figure 2 are also presented. Pearson's correlation for the data set is shown for each endogenous reference gene and the same information is summarized graphically at the bottom of the figure. MEF = mean equivalent of fluorescence [35].

We tested whether gp100 / endogenous reference gene _Log2 _ratios correlated with gp100 protein expression level as measured by intra-cellular FACS analysis (Figure 4). The gp100 / endogenous reference gene _Log2 _ratios were derived from the transcriptome arrays while protein expression was based on previous characterizations of the same cell lines [35,39]. Overall, a good correlation (Pearson's correlation) was noted for gp100 values normalized with most endogenous reference genes. However, with the exception of NEDD8, the best correlation was obtained when pooled information was used from the best 5 and 3 candidate endogenous reference genes as defined before. Interestingly, RAB3 that scored very high as a predictor of gp100 transcriptional expression in melanocytic lesions and β-actin provided the worst correlation with protein expression. This data suggest that, although different endogenous reference genes may yield better predictive value than others in large data sets, it is likely that their reliability varies in different conditions and possibly the best results can be obtained by pooling information from several of them.

**Figure 5:**
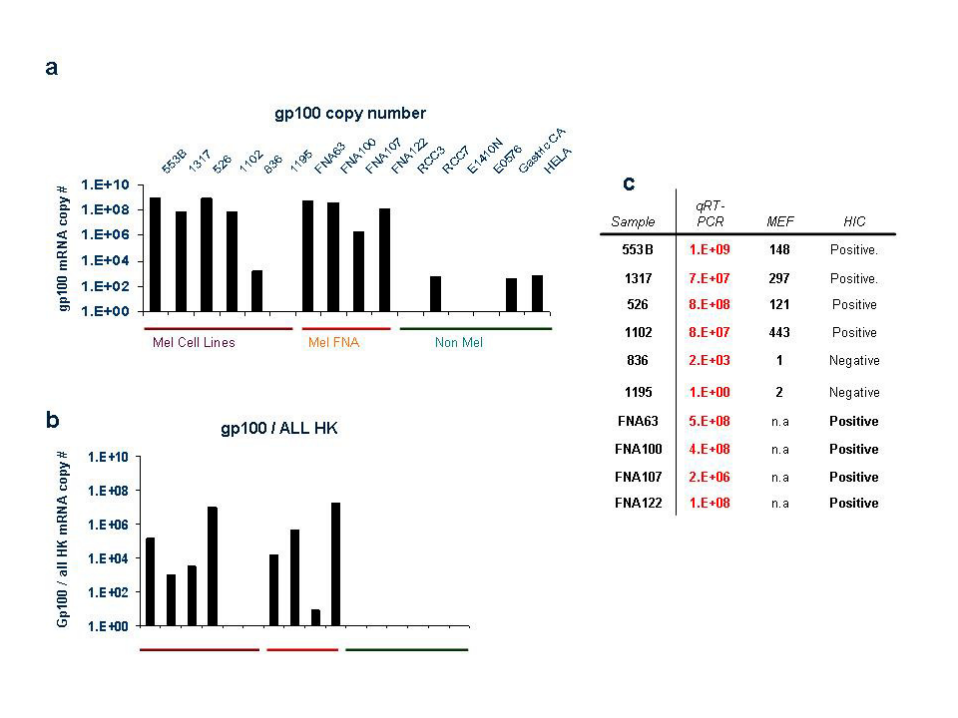
Absolute and normalized expression of gp100 based on qRT-PCR. Four melanoma cell lines expressing gp100 (MEL-553B, MEL 1317, MEL 526 and MEL-1102) and 2 not expressing gp100 (MEL-836 and MEL-1195) protein by FACS analysis [35] (maroon horizontal bar) were tested for gp100 expression by qRT-PCR as previously described [40]. In addition four fine need aspirates of melanoma metastases all expressing GP100 by immunohistochemistry (orange horizontal bars) and two renal cell, two esophageal and one gastric cancer specimens all not expressing gp100 (green horizontal bar) were tested. Similarly the gp100 not expressing HeLa cell line was tested. Absolute gp100 copy number is shown in (a); normalized expression of gp100 is shown using the average of all endogenous reference gene results (b). The protein expression for the melanoma cell lines is presented as mean equivalent of fluorescence (MEF) [35] as well as immunohistochemical evaluation while the expression by FNA is shown in (c) based on immunohistochemical (HIC) evaluation as previously described [46]. Absolute copy numbers of gp100 estimated by qRT-PCR are also shown.

We also tested by quantitative real-time PCR (qRT-PCR) the discriminatory capacity of endogenous reference genes in samples with known patterns of expression of gp100. We selected 6 previously characterized [35,36] melanoma cell lines four of which were known to express (MEL-553B, MEL-1317, MEL-526, MEL-1102) and two known not to express (MEL-836, MEL-1195) gp100 at the transcriptional and protein level (Figure 5) [36,39]. In addition, 4 fine needle aspirate (FNA) samples from melanoma metastases expressing gp100 [40] and a series of tumors (2 renal cell, 2 esophageal one gastric carcinomas and HELA cells line) known not to express gp100 were tested. Absolute expression of gp100 was measured and although the lesions supposed to be gp100 expressing appeared more positive than those supposed to be gp100 negative there was no absolute demarcation among the two groups (Figure 5a). A better differentiation between positive and negative lesions could be obtained when the various endogenous reference genes were used for the analysis or the combined values from all of them (Figure 5b).

## Discussion

Although the concept of endogenous reference genes is appealing it may be unrealistic to expect that a gene could be equally expressed in all eukaryotic cells in all physiopathological conditions. Thus, the endogenous reference gene concept can only approximate such ideal biological behavior. In fact, numerous studies noted that the expression of the ostensible endogenous reference genes varies according to distinct physio-pathological situations and can be affected by experimental manipulation [2,20,24,26,27,41]. For instance, the levels of expression of GAPDH, cyclophilin and β-actin fluctuate in different tissues, disease stage and are affected by cell behavior like proliferation [6,15,18,20,42]. In addition, endogenous reference genes expression varies markedly in cancer compared to normal tissue [17,26,41] suggesting that oncogenesis may influence their expression [25].

In this study, therefore, we looked for relative stable endogenous reference gene challenging a 17,000 cDNA clone data base against approximately 400 specimens that included a broad variety of normal and neoplastic tissues as well as cell lines. Indeed, few previously unnamed genes approximated the ideal endogenous reference gene by being expressed consistently in more than 95% of the specimens analyzed within a variance that was barely and insignificantly different from what could be expected from intrinsic experimental fluctuation (Table 1). In fact, the threshold that we selected based on the variance observed by periodically repeating identical experiments was consistent with criteria suggested by others [43] who claimed that genes with fold changes greater than 1.5 can be classified as differentially expressed at ~95% confidence interval. This is consistent to our selection criterion that contained endogenous reference genes within a 1.46 fold maximal variation. Although these genes have not been proposed before as candidate endogenous reference genes, analysis of available data bases suggested that they are relatively stably expressed in various normal tissues (i.e. Affymetrix HG-U95A-E probe sets). Selection of these candidate endogenous reference genes provided useful information when the expression of a lineage specific gene such as gp100 was compared between relevant and irrelevant samples. In addition, gp100 expression normalized according to some of the new endogenous reference genes more closely aligned to lineage specificity than normalization performed using β-actin.

This data, however, confirm that there is no such thing as a perfect endogenous reference gene. Although some endogenous reference genes demonstrated high power of discrimination their ranking was dictated by previous knowledge of the expected experimental results (gp100 being melanocytic lineage specific). However, gene expression analysis is done to determine such specificity and in most cases no previous knowledge of tissue specificity is truly available. One may argue that, in spite of the extreme variability in normalization power demonstrated by various endogenous reference genes, still significant differences could extracted between the melanocytic and non-melanocytic samples no matter what endogenous reference genes were used. This statement is correct in this experimental situation where hundreds of samples could be compared with the transcriptome array. However, in cases when only few samples are analyzed the difference in discriminatory capacity becomes critical. For instance, when randomly selected gp100 / endogenous reference gene _Log2 _ratios from 10 melanoma metastases were compared with those from 10 kidney specimens only NEDD8, RAb3, FZD6, AFAP, COL8, SNRPD and pooled endogenous reference gene-based normalizations yielded significant differences between the two groups while non significant values were obtained for normalizations based on HIRIP5, β-actin and GPLD1.

The lowest limit of detection in the transcriptome array of specific probes in term of copy numbers is currently under investigation. Decreasing concentrations of β-actin alone printed the array spots as internal controls suggest that the detection range is from 30 ng / spot to 3 pg / spot when standard fluorescent intensity range parameters are applied. The higher limit of detection before saturation can be easily adjusted to avoid under estimates of highly expressed genes, therefore, increasing the dynamic range of detection. This is achieved by adjusting the fluorescent intensity right below the saturation for both channel and subsequently calculating the abundance of reference gene expression using serially diluted internal control spots as standard curve. With few exceptions, the expression of most genes can be estimated with relative accuracy using this semi-quantitative approach.

Since in most cases endogenous reference genes are selected based on previous knowledge of their usefulness for a particular experimental situations, it is likely that the best consistency could be achieved in such circumstances when averages of several endogenous reference gene normalizations are used. This may represent a reasonable solution particularly if a cocktail of endogenous reference genes identically labeled could be used as reference within a single experiment. We are presently testing various combinations of lineage specific genes and pools of endogenous reference genes for hybridization to the transcriptome array and for qRT-PCR. Such attempts, however, have been quite disappointing so far as results obtained using mixture of endogenous reference genes do not yield information comparable to that achievable when results obtained with the same endogenous reference genes used individually are pooled together. This is possibly due to imbalanced density of various endogenous reference genes in different samples with predominant effects of some over others. Therefore, extensive work in the future will be aimed at equilibrating the interactions among endogenous reference genes in various experimental conditions to test whether mixtures of them could be used in multiplex for normalization of large expression analysis studies.

## Conclusions

Our observations, together with previous work by others indicate that much caution should be taken when using endogenous reference to normalize the expression of test genes. It is likely that previous analyses based on single endogenous reference genes might have been strongly biased by the individual selections and the interpretation of the results should looked at with caution. In particular, this study suggests that even when the most stably expressed genes in array experiments are used as endogenous reference genes, significant variation in test gene expression estimates may occur and the best normalization is achieved when data from several endogenous reference genes are pooled together to minimize minimal but significant variation among samples. We are presently optimizing strategies for the preparation of endogenous reference gene mixtures that could yield information comparable to that of data pooled from individual endogenous reference gene normalizations.

## Methods

The list of samples used for cDNA microarray hybridization or for preparation of the transcriptome array is available upon request. Most melanoma and renal carcinoma cell lines were generated at the Surgery Branch, National Cancer Institute (NCI), National Institutes of Health (NIH), Bethesda, MD and maintained in RPMI (Biosource International, Camarillo, CA) supplemented with 10% fetal calf serum (Biosource). Cell lines were harvested by Trypsin /Versene (Biosource) digestion. PBMC were collected by leukapheresis from six unrelated normal donors in the Department of Transfusion Medicine, Clinical Center, NIH and purified by Ficoll gradient separation. Surgically removed tumor tissues were collected from the Tissue Network (Philadelphia, PA), Surgery Branch and CPSB specimen bank, NCI, (Bethesda, MD). Fine needle aspiration (FNA) biopsies were obtained from patients with metastatic melanoma referred to the Surgery Branch, NCI for immunotherapy. Total RNA was isolated using Qiagen RNeasy kit and its quality and quantity was estimated using Agilent Bioanalyzer 2000 (Agilent Technologies, Palo Alto, CA).

### Target preparation for cDNA microarray hybridization

Total RNA extracted from test and reference samples was transcribed *in vitro *into anti-sense RNA (aRNA) and reverse-transcribed into fluorescence labeled cDNA for hybridization to 17,000 gene cDNA-based microarrays [30,44]. Pooled PBMC were used to prepare reference aRNA to be co-hybridized in all experiments with test aRNA. cDNA targets were labeled with Cy3 (green) for reference material and Cy5 (red) for test material with the exception of reciprocal experiments.

### cDNA Microarrays

cDNA (UniGene cluster) microarrays were printed at the Immunogenetics Section, DTM, CC, NIH with a configuration of 32 × 24 × 23 containing 17,500 elements. Clones used for printing were selected from the Research Genetics RG_HsKG_031901 8 k clone set and the 9k RG_Hs_seq_ver_070700 40 k clone set. The 17,500 spots included 12,072 uniquely named genes, 875 duplicate genes and about 4,000 expression sequence tags. For a complete list of genes included in the Hs-CCDTM-17.5k-1px printing please visit our web site at .

### Transcriptome array

A collection of aRNA-based libraries was prepared from 960 frozen tissue samples or cell lines and individual aRNAs were reverse transcribed into cDNA in the presence of 1 μl of dN6 primer (8 μg/μl), 6 μl of first strand buffer, 3 μl of 10 mM dNTP, 3 ul of 50 mM dTT, 1.5 μl of Rnasin and 10 μl of aRNA (6–12 ug) with addition of 3 μl of Superscript II (BRL) in 5.5 μl volume after heating to 65°C for 5 minutes. Reactions were carried out in 96-well plates at 42°C for 90 minutes followed with addition of 1 μl Rnase H and heating for 20 min at 37°C. cDNA were further purified by utilizing CentriCept gelfitration plates to remove non incorporated primer and dNTP. Purified cDNA were transferred to 384-well plates and dried by speedvac. Samples were re-suspended in 13 μl of 3 × SSC followed by shaking at 3,000 rpm for 15 min. Reconstituted cDNA libraries from individual samples and spiked reference gene at different concentration were printed on to poly-L-lysine coated glass slides at the concentration of 0.5–1 μg/μl using the OmniGrid (GeneMachine. San Carlos, CA) printer and Telechem printing pins (TeleChem International, Inc. Sunnyvale, CA). Each 100 μm diameter spot was duplicated at a 250 μm distance. After complete exsiccation, slides were post-processed as described at .

### Probe design Transcriptome array hybridization experiments

Specific PCR primers for the amplification of each gene probe were designed using Primer 3 program . In order to generate single strand fluorescence labeled cDNA for hybridization, modified specific 5' primers with an extension of the T7 promoter region were used for PCR amplification. Double strand PCR fragments (300–800 bp) were used as template for *in vitro *transcription to generate single stranded mRNA. 3 μg of amplified RNA were then reverse transcribed into cDNA in present of 4 μl of first strand buffer, 1 μl dN6 primer (8μg/μl; Boehringer Mannheim), 2 μl 10 X low T-dNTP (5 mM A, C and GTP, 2 mM dTTP), 2 μl Cy-dUTP (1 mM, Cy3 or Cy5), 2 μl 0.1 M DTT, 1 μl RNasin, 3 μg amplified mRNA in 8 μl DEPC H_2_O. After heating to 65°C for 5 minutes and cooling to 42°C, 2 μl of SSII was added and the labeling reaction was carried out at 42°C for 90 min. Probe purification and hybridization were performed as previously described [30,44].

### Quantitative real-time polymerase chain reaction (RT-PCR)

Primer and probe sets from each candidate genes were designed using the Primer Express 2.0 program (Applied Biosystems, Foster City, CA) and synthesized by BioSource. Taqman probes were labeled at the 5' end with the reporter dye molecule 6-carboxy-fluorescein (FAM; emission λ_max _= 518 nm) and at the 3' end with the quencher dye molecule 6-carboxytetramethyl-rhodamine (TAMRA; emission λ_max _= 582 nm). Total RNA was used for gene expression validation. Measurement of gene expression was performed using the ABI 7900HT sequence detection system (Perkin Elmer, Foster City, CA) as previously described [40,45]. Messenger RNA from test samples were reverse transcribed into cDNA in the presence of oligo dT (12–18 mer). The qRT-PCR procedure was performed by alternating 2 minute cycles at 50°C and 10 minute at 95°C. Forty cycles involving denaturation at 95°C for 15 seconds and annealing/extension at 60°C for 1 minute in 50 μl volume with 1 × TaqMan Master Mix (PerkinElmer). Standard curves were generated for each gene with high and accurate PCR amplification efficiency as determined by the slope of the standard curves. Linear regression analysis of all standard curves documented in all cases an R value ≥ 0.99. The sequence of primer sets and probes used for each gene are shown in Table 3 with statistics for each standard curve. Standard curve extrapolation of copy number was performed for the gene of interest as well as an endogenous reference gene for each sample. To correct for concentration of starting material, normalization of samples was performed by dividing the copies of the gene of interest by copies of the reference gene as previously described [40].

**Table 3:** Quantitative RT-PCR primers and probes

AFAP	(+)-TGTCAAGTTAAACCACTAATGTGTTGGT	***Y *= -3.105x + 43.726 R2 = 0.99**
	(-)-GGCATCCAAATTCTCCAAGAAA	
	FAM-TGCTGCCTCTCCTGAGTAGGGTGGGT-TAMRA	
CDK5R1	(+)-TCCTACATGGGCAACGAGATC	***Y *= -3.315x + 45.983 R2 = 0.99**
	(-)-CCAAAAGGCCTCCTTGCA	
	FAM -TACCCGCTCAAGCCCTTCCTGGTG -TAMRA	
COL8A1	(+)-CCGAGCTAACCGCACCTTT	***Y *= -3.143 x +43.294 R2 = 0.99**
	(-)-GTCTGCGGGTTGTAGTTCTGTCT	
	FAM -AGTGAAGTTTAACAAACTGCTGTATAACGG -TAMRA	
FZD	(+)-AGCCTCAAAGGTTCCACATCTC	***Y *= -3.515 x + 47.092 R2 = 0.99**
	(-)-AGGTCACTTCCAGTGTAACACAAATT	
	FAM -TGAGAAAAGAGCAGGGAGGTGGTTGTCA -TAMRA	
GPLD1-1	(+)-CAGATTGAAGATTTCACTGCATTTC	***Y *= -3.653 x + 49.086 R2 = 0.99**
	(-)-CATCAAAATGCTCACCATGGA	
	FAM -TCTGCCCACCTCTCTCATGCTGAATCAC -TAMRA	
GPLD1-2	(+)-CTTTTGCCTGTAGTAGTAAATTGCTTTTA	***Y *= -3.168x + 46.599 R2 = 0.99**
	(-)-AACTGGCCATATAAC CAAAGGTGTT	
	FAM -TGAATGGTGTTTATTAAACCCTTATGGTCGATATTTCC-TAMRA	
HIRIP5	(+)-GCTGCCCTAGTTCAATCATTACTCT	***Y *= -3.160x + 43.313 R2 = 0.99**
	(-)-CGCCTTCTACCTCCGGAATAT	
	FAM – AAAAATGGAATTCAGAACATGCTGCA-TAMRA	
HIST1HIA	(+)-AGGCGTCCTCCGTGGAA	***Y *= -3.143x + 43.294 R2 = 0.99**
	(-)-ATGCACCCGTTGCCTTAGTT	
	FAM – AGCCCGGCGCCTCAAAGGTG-TAMRA	
NEDD8	(+)-TGACCGGAAAGGAGATTGAGA	***Y *= -3.458x + 48.686 R2 = 0.99**
	(-)-CCACACGCTCCTTGATTCG	
	FAM – TGACATTGAACCTACAGACAAGGTGGA-TAMRA	
PTH	(+)-GCATAACCTGGGAAAACATCTGA	***Y *= -3.063x + 45.215 R2 = 0.99**
	(-)-TGCACATCCTGCAGCTTCTT	
	FAM -TCGATGGAGAGAGTAGAATGGCTGC-TAMRA	
RAB31	(+)-CCCCTGAAGGATGCTAAGGAA	***Y *= -3.303x + 48.517 R2 = 0.99**
	(-)-AGCATTTTTTGCACTTGTCTCAAC	
	FAM – ACGCTGAATCCATAGGTGCCATC-TAMRA	
SNRPD3	(+)-GGCACAGCTGGAGCAGGTAT	***Y *= -3.177x + 47.200 R2 = 0.99**
	(-)-CTTCAGCATGTCAGGCAAAATC	
	FAM – ATCCGTGGCAGCAAAATCCGC -TAMRA	
β-actin	(+)-GGCACCCAGCACAATGAAG	***Y *= -3.127x + 44.733 R2 = 0.99**
	(-)-GCCGATCCACACGGAGTACT	
	FAM-TCAAGATCATTGCTCCTCCTGAGCGC-TAMRA	
gp100	(+)GGTTCCTTTTCCGTCACCCT	***Y *= -3.282x + 45.987 R2 = 0.99**
	(-)CTCACCGGACGGCACAG	
	FAM-ACATTGTCCAGGGTATTGAAAGTGCCGAGAT-TAMRA	

## Authors' contributions

PJ validated the system combining high-throughput data with gene-directed analysis by quantitative real-time PCR. YZ performed statistical analysis. YN, MCP, DN, VM and KS participated in the preparation and utilization of array experiments used for the identification of stably expressed genes. NH, HS and PRT provided a large library of samples necessary for the development of the transcriptome array. FMM supervise the project as principal investigator.

EW provided leadership for the development of cDNA arrays, transcriptome array and for the overall conduct of the experiments.
